# 4-{[(5-Methyl-2-fur­yl)methyl­ene]hydrazinocarbon­yl}pyridinium chloride monohydrate

**DOI:** 10.1107/S1600536809028426

**Published:** 2009-07-25

**Authors:** Li-Min Li, Fang-Fang Jian, Li Liu

**Affiliations:** a Microscale Science Institute, Department of Chemistry and Chemical Engineering, Weifang University, Weifang 261061, People’s Republic of China; b Microscale Science Institute, Weifang University, Weifang 261061, People’s Republic of China

## Abstract

The title compound, C_12_H_12_N_3_O_2_^+^·Cl^−^·H_2_O, was prepared by the reaction of *N*′-[(5-methyl-2-fur­yl)methyl­ene]isonicotino­hydrazide and hydro­chloric acid at room temperature. The  entire molecule is approximately planar with a maximum deviation of 0.047 (2) Å. An intramolecular C—H⋯O interaction is observed. O—H⋯Cl, N—H⋯Cl, N—H⋯O, N—H⋯N, C—H⋯Cl and C—H⋯O hydrogen-bonds stabilize the crystal structure.

## Related literature

Schiff bases have been used extensively as ligands in the field of coordination chemistry, see: Cui *et al.* (2005 ▶). For their anti­microbial and anti­cancer applications, see: Tarafder *et al.* (2000 ▶) and Deschamps *et al.* (2003 ▶), respectively.
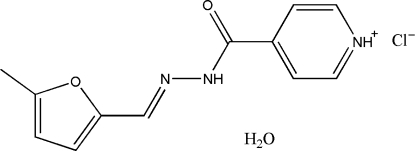


## Experimental

### 

#### Crystal data

C_12_H_12_N_3_O_2_^+^·Cl^−^·H_2_O
*M* *_r_* = 283.71
Monoclinic, 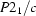

*a* = 8.5258 (17) Å
*b* = 14.435 (3) Å
*c* = 13.625 (4) Å
β = 123.55 (2)°
*V* = 1397.5 (7) Å^3^
*Z* = 4
Mo *K*α radiation
μ = 0.28 mm^−1^
*T* = 293 K
0.20 × 0.15 × 0.11 mm

#### Data collection

Bruker P4 diffractometer
Absorption correction: none
13328 measured reflections
3187 independent reflections
2715 reflections with *I* > 2σ(*I*)
*R*_int_ = 0.026

#### Refinement

*R*[*F* ^2^ > 2σ(*F* ^2^)] = 0.038
*wR*(*F* ^2^) = 0.107
*S* = 1.07
3187 reflections
180 parameters
H atoms treated by a mixture of independent and constrained refinement
Δρ_max_ = 0.27 e Å^−3^
Δρ_min_ = −0.21 e Å^−3^

### 

Data collection: *SMART* (Bruker, 1997 ▶); cell refinement: *SAINT* (Bruker, 1997 ▶); data reduction: *SAINT*; program(s) used to solve structure: *SHELXS97* (Sheldrick, 2008 ▶); program(s) used to refine structure: *SHELXL97* (Sheldrick, 2008 ▶); molecular graphics: *SHELXTL* (Sheldrick, 2008 ▶); software used to prepare material for publication: *SHELXTL*.

## Supplementary Material

Crystal structure: contains datablocks global, I. DOI: 10.1107/S1600536809028426/at2845sup1.cif

Structure factors: contains datablocks I. DOI: 10.1107/S1600536809028426/at2845Isup2.hkl

Additional supplementary materials:  crystallographic information; 3D view; checkCIF report

## Figures and Tables

**Table 1:** Hydrogen-bond geometry (Å, °)

*D*—H⋯*A*	*D*—H	H⋯*A*	*D*⋯*A*	*D*—H⋯*A*
O1*W*—H2*W*1⋯Cl1^i^	0.86 (3)	2.40 (3)	3.229 (3)	162 (3)
O1*W*—H1*W*1⋯Cl1^ii^	0.72 (3)	2.51 (3)	3.225 (3)	177 (2)
N2—H2*A*⋯Cl1^i^	0.86	2.39	3.2243 (15)	164
N3—H3*A*⋯O2^ii^	0.86	1.89	2.639 (2)	144
N3—H3*A*⋯N1^ii^	0.86	2.50	3.2238 (18)	142
C3—H3*B*⋯Cl1^iii^	0.93	2.76	3.6574 (19)	162
C6—H6*A*⋯Cl1^i^	0.93	2.69	3.5374 (18)	151
C9—H9*A*⋯Cl1^i^	0.93	2.64	3.5656 (18)	171
C11—H11*A*⋯O1^ii^	0.93	2.45	3.1694 (19)	135
C12—H12*A*⋯O2	0.93	2.39	2.713 (2)	100

Symmetry codes: (i) 
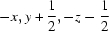
; (ii) 
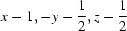
; (iii) 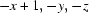.

**Fig. 1.**
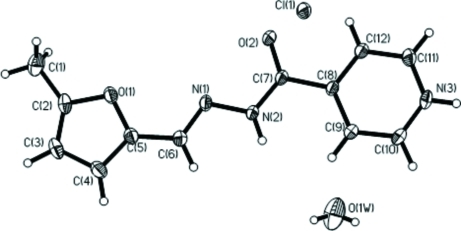
The structure of the title compound showing 30% probability displacement ellipsoids and the atom-numbering scheme.

## supplementary crystallographic information

### Comment

Schiff bases have been used extensively as ligands in the field of coordination chemistry (Cui *et al.*, 2005). And they have antimicrobial (Tarafder *et al.*, 2000) and anticancer applications (Deschamps *et al.*, 2003). The title compound (I) was synthesized and we report its crystal structure here.

In the crystal structure of (I) (Fig. 1), the carbon and nitrogen atoms are nearly the same plane with a maximum deviation of 0.047Å for N2. There are intra- and intermolecular O—H···Cl, N—H···Cl, N—H···O, N—H···N, C—H···Cl and C—H···O hydrogen-bonds to stabilize the crystal structure (Table 1).

### Experimental

A mixture of *N*'-[(5-methyl-2-furyl)methylene]isonicotinohydrazide (0.02 mol) and hydrochloric acid (0.01 mol) was stirred with ethanol (50 ml) at 298 K for 2 h, then afford the title compound (2.61 g, yield 92%). Single crystals suitable for X-ray measurements were obtained by recrystallization from ethanol and trichloromethane (1:1) at room temperature.

### Refinement

The H atoms of the water molecule were found from a difference Fourier map and refined freely. The other H atoms were fixed geometrically and allowed to ride on their attached atoms, with C—H and N—H distances of 0.93–0.96 and 0.86 Å, and with *U*_iso_ = 1.2–1.5*U*_eq_(C,N).

### Crystal data

**Table:**

C_12_H_12_N_3_O_2_^+^·Cl^−^·H_2_O	*F*(000) = 592
*M**_r_* = 283.71	*D*_x_ = 1.348 Mg m^−^^3^
Monoclinic, *P*2_1_/*c*	Mo *K*α radiation, λ = 0.71073 Å
Hall symbol: -P 2ybc	Cell parameters from 2715 reflections
*a* = 8.5258 (17) Å	θ = 3.1–27.5°
*b* = 14.435 (3) Å	µ = 0.28 mm^−^^1^
*c* = 13.625 (4) Å	*T* = 293 K
β = 123.55 (2)°	Bar, yellow
*V* = 1397.5 (7) Å^3^	0.20 × 0.15 × 0.11 mm
*Z* = 4	

### Data collection

**Table:**

Bruker P4 diffractometer	2715 reflections with *I* > 2σ(*I*)
Radiation source: fine-focus sealed tube	*R*_int_ = 0.026
graphite	θ_max_ = 27.5°, θ_min_ = 3.1°
Detector resolution: 3 pixels mm^-1^	*h* = −10→11
ω scans	*k* = −18→18
13328 measured reflections	*l* = −17→17
3187 independent reflections	

### Refinement

**Table:**

Refinement on *F*^2^	Primary atom site location: structure-invariant direct methods
Least-squares matrix: full	Secondary atom site location: difference Fourier map
*R*[*F*^2^ > 2σ(*F*^2^)] = 0.038	Hydrogen site location: inferred from neighbouring sites
*wR*(*F*^2^) = 0.107	H atoms treated by a mixture of independent and constrained refinement
*S* = 1.07	*w* = 1/[σ^2^(*F*_o_^2^) + (0.0543*P*)^2^ + 0.3029*P*] where *P* = (*F*_o_^2^ + 2*F*_c_^2^)/3
3187 reflections	(Δ/σ)_max_ < 0.001
180 parameters	Δρ_max_ = 0.27 e Å^−^^3^
0 restraints	Δρ_min_ = −0.21 e Å^−^^3^

### Special details

**Table:**

Geometry. All e.s.d.'s (except the e.s.d. in the dihedral angle between two l.s. planes) are estimated using the full covariance matrix. The cell e.s.d.'s are taken into account individually in the estimation of e.s.d.'s in distances, angles and torsion angles; correlations between e.s.d.'s in cell parameters are only used when they are defined by crystal symmetry. An approximate (isotropic) treatment of cell e.s.d.'s is used for estimating e.s.d.'s involving l.s. planes.
Refinement. Refinement of *F*^2^ against ALL reflections. The weighted *R*-factor *wR* and goodness of fit *S* are based on *F*^2^, conventional *R*-factors *R* are based on *F*, with *F* set to zero for negative *F*^2^. The threshold expression of *F*^2^ > σ(*F*^2^) is used only for calculating *R*-factors(gt) *etc*. and is not relevant to the choice of reflections for refinement. *R*-factors based on *F*^2^ are statistically about twice as large as those based on *F*, and *R*- factors based on ALL data will be even larger.

### Fractional atomic coordinates and isotropic or equivalent isotropic displacement parameters (Å^2^)

**Table:**

	*x*	*y*	*z*	*U*_iso_*/*U*_eq_	
Cl1	0.22892 (6)	−0.42533 (3)	−0.18006 (4)	0.06023 (16)	
O1	0.51022 (13)	0.04123 (6)	0.13453 (8)	0.0380 (2)	
O2	0.05971 (14)	−0.22008 (7)	−0.02944 (10)	0.0536 (3)	
N1	0.16922 (14)	−0.04732 (7)	−0.03026 (9)	0.0327 (2)	
N2	−0.00554 (14)	−0.08118 (7)	−0.11953 (9)	0.0327 (2)	
H2A	−0.0844	−0.0467	−0.1778	0.039*	
N3	−0.57485 (15)	−0.28167 (8)	−0.36865 (9)	0.0392 (3)	
H3A	−0.6822	−0.3057	−0.4198	0.047*	
C1	0.8247 (2)	0.07121 (14)	0.30399 (16)	0.0616 (5)	
H1B	0.9141	0.1209	0.3397	0.092*	
H1C	0.8779	0.0215	0.2847	0.092*	
H1D	0.7941	0.0493	0.3580	0.092*	
C2	0.65193 (19)	0.10503 (11)	0.19547 (13)	0.0419 (3)	
C3	0.5985 (2)	0.18739 (10)	0.14057 (14)	0.0469 (4)	
H3B	0.6695	0.2415	0.1641	0.056*	
C4	0.4137 (2)	0.17617 (10)	0.04032 (13)	0.0441 (3)	
H4A	0.3395	0.2216	−0.0145	0.053*	
C5	0.36538 (19)	0.08648 (9)	0.03927 (12)	0.0354 (3)	
C6	0.19437 (19)	0.03842 (9)	−0.04240 (11)	0.0359 (3)	
H6A	0.0978	0.0707	−0.1067	0.043*	
C7	−0.04668 (16)	−0.16914 (9)	−0.11160 (10)	0.0321 (3)	
C8	−0.23608 (16)	−0.20547 (8)	−0.20798 (10)	0.0301 (3)	
C9	−0.37266 (17)	−0.15335 (9)	−0.30253 (11)	0.0365 (3)	
H9A	−0.3495	−0.0920	−0.3115	0.044*	
C10	−0.54274 (18)	−0.19394 (10)	−0.38265 (11)	0.0404 (3)	
H10A	−0.6356	−0.1601	−0.4468	0.049*	
C11	−0.4484 (2)	−0.33342 (10)	−0.27924 (13)	0.0441 (3)	
H11A	−0.4768	−0.3942	−0.2721	0.053*	
C12	−0.27438 (19)	−0.29683 (9)	−0.19686 (12)	0.0413 (3)	
H12A	−0.1837	−0.3330	−0.1346	0.050*	
O1W	−0.3179 (3)	−0.06393 (18)	−0.5308 (2)	0.1060 (7)	
H2W1	−0.282 (4)	−0.0189 (19)	−0.481 (2)	0.093 (8)*	
H1W1	−0.419 (4)	−0.0676 (18)	−0.566 (2)	0.084 (9)*	

### Atomic displacement parameters (Å^2^)

**Table:**

	*U*^11^	*U*^22^	*U*^33^	*U*^12^	*U*^13^	*U*^23^
Cl1	0.0599 (3)	0.0341 (2)	0.0513 (2)	−0.00819 (16)	0.00847 (19)	−0.00564 (15)
O1	0.0337 (5)	0.0317 (5)	0.0423 (5)	−0.0068 (4)	0.0170 (4)	−0.0055 (4)
O2	0.0291 (5)	0.0402 (5)	0.0497 (6)	−0.0051 (4)	−0.0044 (4)	0.0125 (5)
N1	0.0253 (5)	0.0331 (5)	0.0312 (5)	−0.0053 (4)	0.0104 (4)	−0.0060 (4)
N2	0.0240 (5)	0.0305 (5)	0.0292 (5)	−0.0022 (4)	0.0056 (4)	−0.0012 (4)
N3	0.0252 (5)	0.0466 (7)	0.0317 (5)	−0.0085 (5)	0.0070 (4)	−0.0110 (5)
C1	0.0368 (8)	0.0758 (12)	0.0565 (10)	−0.0073 (8)	0.0159 (7)	−0.0122 (9)
C2	0.0348 (6)	0.0454 (7)	0.0472 (8)	−0.0143 (6)	0.0236 (6)	−0.0167 (6)
C3	0.0515 (8)	0.0396 (7)	0.0572 (9)	−0.0204 (7)	0.0348 (7)	−0.0155 (7)
C4	0.0531 (8)	0.0334 (7)	0.0484 (8)	−0.0089 (6)	0.0297 (7)	−0.0025 (6)
C5	0.0377 (7)	0.0321 (6)	0.0359 (6)	−0.0053 (5)	0.0200 (6)	−0.0047 (5)
C6	0.0348 (6)	0.0332 (6)	0.0335 (6)	−0.0030 (5)	0.0151 (5)	−0.0032 (5)
C7	0.0221 (5)	0.0321 (6)	0.0305 (6)	−0.0002 (5)	0.0072 (5)	−0.0007 (5)
C8	0.0217 (5)	0.0319 (6)	0.0282 (5)	−0.0003 (5)	0.0084 (5)	−0.0021 (5)
C9	0.0278 (6)	0.0365 (6)	0.0326 (6)	−0.0007 (5)	0.0089 (5)	0.0038 (5)
C10	0.0262 (6)	0.0476 (8)	0.0302 (6)	0.0008 (6)	0.0047 (5)	0.0022 (5)
C11	0.0386 (7)	0.0333 (7)	0.0435 (7)	−0.0094 (6)	0.0120 (6)	−0.0066 (6)
C12	0.0319 (6)	0.0307 (6)	0.0384 (7)	−0.0011 (5)	0.0050 (5)	0.0010 (5)
O1W	0.0743 (12)	0.152 (2)	0.0905 (13)	−0.0102 (12)	0.0446 (11)	−0.0516 (13)

### Geometric parameters (Å, °)

**Table:**

O1—C5	1.3655 (17)	C3—H3B	0.9300
O1—C2	1.3742 (16)	C4—C5	1.3566 (19)
O2—C7	1.2225 (16)	C4—H4A	0.9300
N1—C6	1.2823 (17)	C5—C6	1.4324 (18)
N1—N2	1.3911 (14)	C6—H6A	0.9300
N2—C7	1.3375 (16)	C7—C8	1.5044 (16)
N2—H2A	0.8600	C8—C12	1.3868 (18)
N3—C11	1.3239 (18)	C8—C9	1.3869 (17)
N3—C10	1.3314 (19)	C9—C10	1.3741 (18)
N3—H3A	0.8600	C9—H9A	0.9300
C1—C2	1.479 (2)	C10—H10A	0.9300
C1—H1B	0.9600	C11—C12	1.3780 (18)
C1—H1C	0.9600	C11—H11A	0.9300
C1—H1D	0.9600	C12—H12A	0.9300
C2—C3	1.343 (2)	O1W—H2W1	0.87 (3)
C3—C4	1.412 (2)	O1W—H1W1	0.72 (3)
			
C5—O1—C2	106.56 (11)	C4—C5—C6	130.05 (13)
C6—N1—N2	113.64 (11)	O1—C5—C6	120.28 (11)
C7—N2—N1	117.70 (10)	N1—C6—C5	122.56 (12)
C7—N2—H2A	121.1	N1—C6—H6A	118.7
N1—N2—H2A	121.1	C5—C6—H6A	118.7
C11—N3—C10	122.76 (11)	O2—C7—N2	123.37 (11)
C11—N3—H3A	118.6	O2—C7—C8	119.01 (11)
C10—N3—H3A	118.6	N2—C7—C8	117.61 (10)
C2—C1—H1B	109.5	C12—C8—C9	119.33 (11)
C2—C1—H1C	109.5	C12—C8—C7	116.11 (11)
H1B—C1—H1C	109.5	C9—C8—C7	124.53 (11)
C2—C1—H1D	109.5	C10—C9—C8	118.83 (13)
H1B—C1—H1D	109.5	C10—C9—H9A	120.6
H1C—C1—H1D	109.5	C8—C9—H9A	120.6
C3—C2—O1	110.02 (13)	N3—C10—C9	120.13 (12)
C3—C2—C1	133.87 (14)	N3—C10—H10A	119.9
O1—C2—C1	116.11 (14)	C9—C10—H10A	119.9
C2—C3—C4	106.92 (13)	N3—C11—C12	119.73 (13)
C2—C3—H3B	126.5	N3—C11—H11A	120.1
C4—C3—H3B	126.5	C12—C11—H11A	120.1
C5—C4—C3	106.83 (14)	C11—C12—C8	119.21 (12)
C5—C4—H4A	126.6	C11—C12—H12A	120.4
C3—C4—H4A	126.6	C8—C12—H12A	120.4
C4—C5—O1	109.67 (12)	H2W1—O1W—H1W1	111 (3)

### Hydrogen-bond geometry (Å, °)

**Table:**

*D*—H···*A*	*D*—H	H···*A*	*D*···*A*	*D*—H···*A*
O1W—H2W1···Cl1^i^	0.86 (3)	2.40 (3)	3.229 (3)	162 (3)
O1W—H1W1···Cl1^ii^	0.72 (3)	2.51 (3)	3.225 (3)	177 (2)
N2—H2A···Cl1^i^	0.86	2.39	3.2243 (15)	164
N3—H3A···O2^ii^	0.86	1.89	2.639 (2)	144
N3—H3A···N1^ii^	0.86	2.50	3.2238 (18)	142
C3—H3B···Cl1^iii^	0.93	2.76	3.6574 (19)	162
C6—H6A···Cl1^i^	0.93	2.69	3.5374 (18)	151
C9—H9A···Cl1^i^	0.93	2.64	3.5656 (18)	171
C11—H11A···O1^ii^	0.93	2.45	3.1694 (19)	135
C12—H12A···O2	0.93	2.39	2.713 (2)	100

Symmetry codes: (i) −*x*, *y*+1/2, −*z*−1/2; (ii) *x*−1, −*y*−1/2, *z*−1/2; (iii) −*x*+1, −*y*, −*z*.
